# Socio-demographic variations in prostate cancer diagnosis recording: Primary care compared to the Cancer Registry in England

**DOI:** 10.23889/ijpds.v9i5.2589

**Published:** 2024-09-18

**Authors:** Gayasha Somathilake, Elizabeth Ford, Jo Armes, Sotiris Moschoyiannis, Sophie Otter, Agnieszka Lemanska

**Affiliations:** 1 School of Health Sciences, Faculty of Health and Medical Sciences, University of Surrey; 2 Department of Primary Care and Public Health, Brighton and Sussex Medical School; 3 Department of Computer Science, Faculty of Engineering and Physical Sciences, University of Surrey; 4 Royal Surrey County Hospital; 5 Data Sciences Department, National Physical Laboratory

## Introduction

In the UK, primary care data are often used for cancer-related research, but the accuracy of cancer information is uncertain.

## Objective

We investigated socio-demographic variation based on the recording date of prostate cancer diagnosis between primary care and the National Cancer Registry (CR).

## Approach

We utilised a data extract of 1,600,000 patients over 65 years from Clinical Practice Research Datalink (CPRD). We extracted prostate cancer diagnoses using Read and SNOMED-CT codes from primary care, and ICD-10 from CR. Initial code entry determined diagnosis dates. We categorised recording timing differences as earlier, same-day or later in primary care than CR and used the chi-squared test and logistic regression (adjusted for recording year) to compare these discrepancies across age, deprivation, and ethnicity.

## Results

We included 26,875 men with prostate cancer diagnoses commonly recorded in both sources during 2000-2016 (1,030 excluded with missing ethnicity). Compared to CR, 1,747 (7%) had diagnoses recorded on the same day in primary care, while 20,615 (77%) had later recordings with a median delay of 21 days (IQR: 13-38). Age at diagnosis was associated with recording discrepancies (p<0.001); older men were more likely to have earlier/same-day recordings. Adjusted ORs for age groups were 1.4 (95%CI: 1.3-1.5) for 60-69, 1.3 (1.2-1.5) for 70-79, and 0.9 (0.8-1.0) for ≥80 compared to <60. No associations were found between deprivation (p=0.096) or ethnicity (p=0.067) and recording differences.

## Conclusion/ Implications

The discrepancy in prostate cancer information between primary care and CR underscores potential biases in studies relying solely on one data source.

